# Reasons and Outcomes of Admissions to the Medical Wards of Jimma University Specialized Hospital, Southwest Ethiopia

**DOI:** 10.4314/ejhs.v20i2.69437

**Published:** 2010-07

**Authors:** Elias Ali, Mirkuzie Woldie

**Affiliations:** 1 Department of Pharmacology, Jimma University, P.O. Box 1637 Jimma, Ethiopia, E-mail: eliasaliyesuf@yahoo.com; 2 Department of Health Services Management, Jimma University, P.O. Box 1637, Jimma, Ethiopia, E-mail: mirkuzie@yahoo.com

**Keywords:** Patient, admissions, reasons, medical outcomes

## Abstract

**Background:**

Non-communicable diseases are the main reasons for admission to the medical wards in high-income countries. While in low and middle income countries communicable diseases are the main reasons for admission to the medical wards. However, in some low and middle income countries the reasons for admission are changing from communicable diseases to non-communicable diseases. But, data on reasons for admission to the medical wards of low income countries is scarce. Therefore, this study takes one year data from a low income country referral hospital aiming at describing the recent reasons and outcomes of medical admissions to see whether there is a change in reasons for admission and the outcome.

**Methods:**

A retrospective study examined patient case notes and ward registration books of medical admissions at Jimma University Specialized Hospital from January 1, 2008 to December 31, 2008. Socio-demographic variables, reasons and outcomes of admission were some of the variables recorded during the data collection. The International Statistical Classification of Disease was used for sorting and categorizing the diagnosis. The data was then analyzed using SPSS windows version 13.0.

**Result:**

A total of 610 patient case notes were reviewed. The mean age of the patients was 36 years (SD ± 15.75). The highest number of admissions 218 (35.7%) was among the age groups 21 to 30 years. Communicable diseases; namely severe community acquired pneumonia 139(22.8%), all infectious and parasitic diseases category 100 (16.4%), and pyogenic as well as chronic meningitis 80(13.1%) were the most common reasons for admission. The death rate among patients admitted to the medical wards was 12.6%.

**Conclusions:**

Communicable diseases were still the common reasons for medical admissions at Jimma University Specialized Hospital. The outcome of medical admissions has not changed over sixteen years.

## Introduction

The decision to admit patients to the medical wards is determined by age, co-existing illness (co-morbidity), physical and laboratory findings, the ability of the patient to comply reliably with an oral medication, and the resources available to the patient outside the hospital (1).

In studies done in developed countries, medical admissions accounted for 22.2%, 33.0%, and 13.0% of total hospital admissions in U.S.A, Western Australia and Hong Kong, respectively (2–4). Whereas, in a South African study admissions to the medical wards constituted 40% of the total hospital admissions (5).

In developed countries Non-communicable diseases namely cardiovascular diseases are the main reasons for medical admissions.. For instance, In the Australian study, the most common reason for admissions to the medical wards was cardiovascular disease, 29% (3). In another study, admissions to medical wards at a hospital in Hong Kong were most frequently associated with the cardiovascular system which made up 30.3% of all medical cases (4). However, in cities and towns of developing countries, the increasing urbanization and westernization of the population is changing the morbidity pattern of diseases (6–9). It is becoming widely accepted that non-communicable or chronic diseases are also now the major causes of death and disability in low and middle income countries (10–12). Despite a decrease in life expectancy because of the HIV epidemic in some Sub-Saharan African countries (13), populations are ageing in low and middle income countries. Moreover, health care to prevent and control these diseases is expensive and unavailable, and the inhabitants are increasingly exposed to risk factors (14). In particular, smoking is increased in underdeveloped countries. The annual cigarette consumption per adult (in cigarettes) has increased from 860 in the early 1970s to 1410 in 1995. The reason was aggressive marketing of tobacco companies, delay in implementing antismoking regulations, and because of the public perception of the risk of smoking is still low (15).

This has been supported by studies from South Africa. Circulatory disorders (22%) and infectious diseases (19%) were shown to be the main causes for admission to medical wards of GF Jooste Hospital (5). In another study from the same country medical admissions were mostly associated with the circulatory system (27.9%) followed by respiratory (15.9%) and infectious diseases (11.9%) (8).

Unlike the findings in developed and some Sub-Saharan African countries, the leading reasons for admission to the medical wards in Ethiopia were found to be communicable diseases. Particularly, communicable diseases such as acute febrile illness of infectious origin, pneumonia and tuberculosis were the reasons for admisson to the medical wards (16).

Referral hospitals are often a highly specific focal point for disease-specific health promotion and education (17). Even though review of records of referral hospital admissions may not indicate the actual prevalence of diseases in the community; it will provide clues about the changing pattern of diseases affecting the community. Unfortunately, data on the specific diseases that indicate reasons for admission to referral hospitals in Ethiopia and their outcomes is scarce. In a study which was undertaken 16 years ago a 2 year record (between Sep 1993 and August 1995) of admission to the medical wards of Jimma University Specialized hospital (JUSH) was reviewed. This study found infectious diseases as the major reasons for admission and a high mortality rate of 12.3% (16). But this study lacks the assessment of complications which are very important in understanding the mortality in hospitals. In addition, this study didn't determine the relationship between the reasons for admission and socio-demographic variables such as sex, and place of residence. It also lacked the assessment of outcomes in relation to hospital stay. Therefore, the objective of this study was to describe the reasons and outcomes of admissions to the medical wards of JUSH.

## Methods

A retrospective cross-sectional study was conducted from May 16 to May 26, 2009 on patients who were admitted to the medical wards of Jimma University Specialized Hospital from January 1, 2008 to December 31, 2008. The hospital serves about 11 million people living within a very wide catchment area of about 250 km radius. It is a training center for about 700 health sciences students each year. The hospital has four major (Medical, Surgery, Gynecology/obstetrics, and pediatrics) and five other departments. The hospital provides postgraduate training in Internal Medicine, Surgery, Gynecology/ obstetrics, Pediatrics, and Ophthalmology. It has 450 beds and a total of more than 550 employees. Internal medicine department has 67 beds. The main diagnostic modalities in the hospital are routine laboratory investigations, radiology and histo-pathologic techniques.

All patients who were admitted to the medical wards of JUSH during the study period (January.1, 2008 to December. 31, 2008) and whose case notes were available in the hospital registration room archive were included. Case notes were classified according to year of admission. The recent case notes of patients admitted to the medical wards in the year 2008 were retrieved. Hence, all cases found during the study period were included in the study and no sampling technique was used. The dependent variables in this study were reasons for admission and outcome of admission, while the explanatory variables included socio-demographic characteristics of the patients in the case notes reviewed, co-morbidities, complications, duration of hospital stay and month of admission.

During data collection case notes of patients admitted to the medical wards and ward registration book were reviewed by two nurses. The data collectors were oriented on completing the structured data collection format prepared for the study. The data collection format included socio-demographics of the respondents, the reason for admission, the co-morbidities, duration of hospital stay, and complications where they develop in the hospital or present from the outset. The format also included outcome variables like discharge with improvement, transfer to other wards, referral to other hospitals, and death. These outcome variables were documented from the registration book and then sorted using International Classification of Diseases (ICD) ten version 2007 (18). Patient case notes with incomplete information were excluded from the study. Then the data obtained was entered into SPSS version 13.0 and analysis was done to obtain descriptive measures. Finally, X^2^ testes of association were done at a significance level of 0.05 wherever appropriate.

Before starting data collection letter of support was obtained from the student research program and permission was obtained from the hospital administration. Moreover, confidentiality of personal information was maintained during data collection, analysis, and interpretation. The following operational definitions were used in the study:

- Reason for admission: is the primary diagnosis given to the illness of the patient by the physician when the patient was admitted.
- Co-morbidity: is an illness which had occurred with the primary diagnosis during the time of admission
- Complications: Severe symptoms of the disease which could lead to death unless treated.
- Season of admission: the particular time (month) of the year during which the patient was admitted.
- Outcome of admission: is diagnosis at discharge.

## Results

A total of 1400 patients were admitted to the medical wards during the one year period. Of these, 614 (44.0%) case notes of patients were available for review. Among these 326 (53.1%) were men and the male to female ratio was 1.1 to 1. The age of the patients ranged from 14 to 90. The mean age of the patients was 36 years (SD ± 15.75). The highest number of admissions came from the age group of 21 to 30 years, 221 (36.0%). The patients were predominantly, 362 (59.0%) from the rural areas (Table 1).

**Table 1:** Socio-demographic characteristics of the patients admitted to JUSH, Southwest Ethiopia, 2008

Sociodemographic variable (n=614)		Number (%)
Sex of the patient		
	Male	326 (53.1%)
	Female	288 (46.9%)
Place of residence		
	Urban	252 (41%)
	Rural	362 (59%)
Age category of the patients		
	14–20	89 (14.5%)
	21–30	221 (36 %)
	31–40	123 (20%)
	41–50	84 (13.7%)
	51–60	49 (8%)
	Above 60	48 (7.8%)

It was found that the most common reasons for admission to the medical wards of JUSH were diseases of the respiratory system, 164 (26.9%) followed by infectious and parasitic diseases, 100 (16.4%), diseases of the nervous system, 88 (14.4%), and diseases of the circulatory system, 178 (12.8%). In the category of the respiratory system diseases the major subcategory were community acquired pneumonia, 139 (84.8%) followed by pneumocystic carinii pneumonia and chronic lower respiratory diseases each accounting for, 9 (5.5%) of the admissions in this category (Table 2).

**Table 2:** Reasons for admission of patients to the medical wards of JUSH, South west Ethiopia, 2008

ICD category*	Reasons for admission		Number	Percent
A or B	Certain infectious and parasitic diseases		100	16.4
		Malaria	52	8.5
		Tuberculosis	33	5.4
		Human immunodeficiency virus (HIV) disease	4	0.7
		Relapsing fever	3	0.5
		Others^†^	8	1.3
C	Malignant Neoplasms		4	0.7
D	Anemia		31	5.1
E	Endocrine, nutritional and metabolic disease		11	1.8
		Diabetes Mellitus	7	1.2
		Others^‡^	4	0.7
G	Diseases of the Nervous system		88	14.4
		Pyogenic and chronic Meningitis	80	13.1
		Polyneuropathies	6	1.0
		Others^§^	2	0.4
I	Diseases of the circulatory system		78	12.8
		Cerebrovascular diseases	20	3.3
		Valvular heart diseases and cardiomyopathies	18	3.0
		Hypertensive diseases	13	2.1
		Ischaemic heart diseases	11	1.8
		Corpulmonale	10	1.6
		Others^‖^	6	1.1
J	Diseases of the respiratory system		164	26.9
		Severe community Acquired Pneumonia	139	22.8
		Pneumocystis carinii pneumonia	9	1.5
		Chronic lower respiratory diseases	9	1.5
		Acute upper respiratory infections	4	0.7
		Others^¶^	3	0.6
K	Diseases of the Digestive system		56	9.2
		Chronic liver disease	22	3.6
		Peritonitis	21	3.4
		Acute gastroenteritis	9	1.5
		Others**	4	0.7
M	Spondylopathies and other MSS diseases		9	1.5
N	Diseases of the genitourinary system		61	10.0
		Renal failure	28	4.6
		Urinary tract infections	17	2.8
		Glomerular diseases	12	2.0
		Others^††^	4	0.7
O	Miscellaneous conditions		12	

* ICD= International Classification of Diseases and related health problems

^†^= protozoal diseases

^§^= malnutrition

^‖^= nerve, nerve root, and plexus disorders not specifically diagnosed

^¶^= acute rheumatic fever, deep venous thrombosis

^¶^= Lung abscess

**= non-infective colitis, acute gastritis, chronic gastritis

^††^= Other Disorders of genito-urinary tract whose diagnoses is not specified

Malaria and tuberculosis were the major subcategories in the infectious and parasitic diseases category accounting for, 52 (52.0%) and 33 (33.0%) of admission in this category, respectively. Moreover, meningitis (mainly pyogenic) is the single most common subcategory, 80 (91.0%) of diseases of the central nervous system (Table 2).

**Figure 1:**
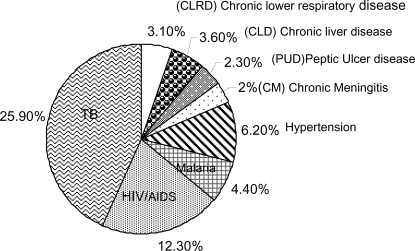
The major co-morbidities of patients admitted to the medical wards of JUSH, Southwest Ethiopia, 2008.

**Figure 2:**
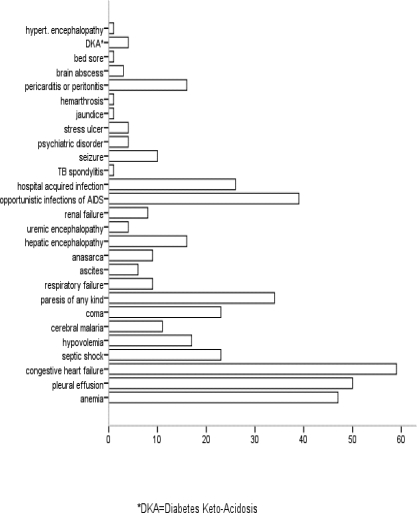
The main complications in patients admitted to the medical wards of JUSH, southwest Ethiopia, 2008.

Regarding the co-morbidities seen at the medical wards, 446 (73.1%) of patients had co-morbidity at admission. The co-morbidities are mainly in the infectious and parasitic diseases category, 264 (59.2%) followed by diseases of the circulatory system, 58 (13.0%) and the digestive system, 42 (9.4%). Tuberculosis alone accounted for 25.9% of the co-morbidities making it the major co-morbidity followed by Human Immune-Deficiency Virus (HIV) infection (12.3%) (Fig. 1). At the time of presentation or following admission 425 (69.7%) of the patients had complications. From these, 360 (84.7%) had only one complication and the remaining 65 (15.3%) patients were found to have more than one complications. The main complications seen at the medical wards include congestive heart failure, pleural effusion, anemia, and opportunistic infections and immune reconstitution inflammatory syndrome (IRIS) of HIV/AIDS each accounting for 59 (9.7%), 50 (8.2%), 46 (7.5%), and 39 (6.4%) of the complications, respectively (Fig. 2)

Regarding the hospital stay of patients, 237 (39.3%) patients stayed in the hospital for five to ten days, 224 (36.9%) for more than ten days, while the remaining 145 (23.8%) had a hospital stay less than five days.

With regard to the outcome of admissions to the medical wards, four hundred and fifty seven (75.0%) patients were discharged with improvement. While 77 (12.6%) patients died in the hospital. Patients who had been discharged with the outcome of the same, self discharge, and referral to other hospitals have together accounted for the remaining 76 (12.4% )of the cases.

The association of sex and place of residence of the patients with the reasons for admission, and the association of hospital stay and the outcome of admission was assessed using chi-square test at a significance level of 0.05.

In the top three major reasons for admission males have out-numbered females except in the infectious and parasitic diseases category. It was also observed that there is a strong association between sex of the patients and the reasons for admission (p-value =0.009) (Table 3).

**Table 3:** Association of the sex of the patients with the reasons for admission to the medical wards of JUSH, South west Ethiopia. 2008

ICD category	Reason for admission	sex		
	
		Male N (%)	Female N (%)	p-value
A or B	Infectious and parasitic disease	40	60	
C	Malignant neoplasms	1	3	
D	anemia	17	22	
E	Endocrine, Nutritional, and Metabolic disease	8	3	
G	Diseases of the nervous system	49	39	
I	Diseases of the circulatory system	54	23	0.007
J	Diseases of the respiratory system	89	75	
K	Diseases of the digestive system	32	24	
M	Spondilopathies and other MSS disorders	5	4	
N	Diseases of the genitourinary system	27	34	
O	Miscellanious conditions	4	1	

	Total	326	614	

X^2^ test value= 25.5

However, no significant association (p=0.951) was found between reasons of admission and place of residence.

Majority of the patients 41 (53.0%) who died in the hospital stayed for less than 5 days in the hospital. It was also found that there is a strong association between hospital stay and outcomes of admission (p-value =0.000) (Table 4).

**Table 4:** Association of hospital stay with the outcome of patients admitted to the medical wards of JUSH, southwest Ethiopia, 2008*

Out come	Hospital stay			
	
	< 5 days	5–10 days	> 10 days	
Improved & discharged	86	188	183	p-value
Same and discharged	5	16	9	=0.000
Self discharge	10	11	9	
Referred	3	5	4	
	
Died	41	17	19	

X^2^ test value =71.23

* some diseases e.g. spondilopathies which have contributed to a very small number of admissions were excluded from this analysis

## Discussion

The results of this study have shown that communicable diseases were the major reasons for medical admissions at JUSH. Besides, the outcomes of medical admissions have not changed over a decade and six years.

The results of this study indicated that patients admitted to the medical wards were mostly between 21 and 30 years (young adults). These results are in line with findings from other studies carried out elsewhere in Ethiopia (16). However, in South African and Nigerian studies, it was observed that patients admitted to the medical wards were mostly people older than 40 years of age (5, 20). This could be explained by the difference in the population composition of the countries.

In contrast to JUSH where communicable diseases are found to be the main reasons for admission, non-communicable diseases are the main reasons for admission at certain referral hospitals of middle income countries. Circulatory disorders are the main reasons for medical admissions at GF Jooste Hospital (South Africa), Queen Mary Hospital (Hong Kong), and Hillbrow Hospital (South Africa) accounting for 22.0%, 40.0%, and 27.9% of medical admissions, respectively (4, 5, 8). The difference from the current study might be due to a change in disease pattern from communicable to non-communicable diseases in the urban areas of developing countries (6–9). In South Africa, unhealthy lifestyles, such as tobacco use, unhealthy nutrition, and lack of regular aerobic physical activity have contributed for increased admissions due to cardiovascular system diseases (21). Such change might be absent in Ethiopia.

The death rate (12.6%) was similar to the findings of a previous study in Jimma Hospital 16 years ago (12.3%) (16). This maintained death rate can partially be explained by the presence of severe complications such as congestive heart failure, hepatic encephalopathy, opportunistic infections of HIV/AIDS among patients admitted to the medical wards. Moreover, in this study 53.0% of patients who died stayed for less than 5 days in the hospital, this early death within few days of admission could be due to the severity of the diseases at the time of presentation to the hospital (16, 20).

The hospital administration and all concerned bodies should note that the death rate in the hospital has not changed over 16 years. Having all the advances in medical care during this period measures should be introduced to ensure that improvements in services provided lead to better outcomes in patients served by the hospital. Moreover, since 75.0% of the patients who died in the hospital have died within 10 days of admission we recommend further study to understand the reasons contributing for the death of patients within 10 days of admission to the medical wards.

Finally, inability to include all case notes due to lose of the documents and including only one year data are the limitations of this study.
